# Erratum to: Histopathological features of bone regeneration in a canine segmental ulnar defect model

**DOI:** 10.1186/s13000-016-0534-y

**Published:** 2016-09-09

**Authors:** Rahim Hobbenaghi, Paria Mahboub, Siamak Saifzadeh, Javad Javanbakht, Javad Yaghoobi Yeganeh Manesh, Rasool Mortezaee, Seyed Rashid Touni, Ehsan Hosseini, Shahin Aghajanshakeri, Milad Moloudizargari, Soheil Javaherypour

**Affiliations:** 1 Department of Pathology, Faculty of Veterinary Medicine, Urmia University, Urmia, Iran; 2 Graduate, Faculty of Veterinary Medicine, Urmia University, Urmia, Iran; 3 Institute of Health and Biomedical Innovation, Queensland University of Technology, 60 Musk Avenue, Kelvin Grove, Brisbane, Qld 4059 Australia; 4 Department of Pathobiology, Faculty of Veterinary Medicine, Tehran University, Tehran, Iran; 5 Gradute of Islamic Azad University of bShahrekord, Faculty of Veterinary Medicine, Shahrekord University, Shahrekord, Iran; 6 Student of Ferdowsi University of Mashhad, Faculty of Veterinary Medicine, Ferdowsi University, Mashahd, Iran; 7 Ph.D Student of Anatomy and Embryology, Faculty of Veterinary Medicine, Urmia University, Urmia, Iran; 8 Faculty of Para Veterinary Medicine, Ilam University, Ilam, Iran; 9 Student of Veterinary Medicine, Faculty of Veterinary Medicine, Urmia University, Urmia, Iran

## Erratum

Unfortunately, an author’s name was spelled incorrectly in the original version of this article [1]. Paria Mahboub’s name has been corrected to reflect this.
